# Clinical and Surgical Implications of Crossed Fusion Renal Ectopia Type E: A Case Report

**DOI:** 10.7759/cureus.107328

**Published:** 2026-04-19

**Authors:** Jarod C Banks, Ellie Burkle, Adam Kolatorowicz

**Affiliations:** 1 Anatomical Sciences, Lincoln Memorial University DeBusk College of Osteopathic Medicine, Knoxville, USA

**Keywords:** cadaver case report, congenital anomalies of the kidney and urinary tract (cakut), crossed fusion renal ectopia, medical education, urologic surgery

## Abstract

Crossed fused renal ectopia (CFRE) is a rare congenital anomaly in which one kidney crosses the midline and fuses with its contralateral counterpart due to abnormal migration during fetal development. Although often asymptomatic, associated anatomical variation may have important clinical and surgical implications. This report describes a rare CFRE Type E (L-shaped) identified during routine anatomical dissection. A 76-year-old male anatomical donor was found to have right-to-left renal ectopy with fusion at the inferior pole of the left kidney. The fused renal unit demonstrated complex vasculature, including five renal arteries and three renal veins with numerous tributaries. Two ureters were present with atypical positioning. The donor’s medical history included hypertension, atrial flutter, hypothyroidism, and aortic insufficiency, with a self-reported history of bilateral pleural effusions, pneumonia, and renal cell carcinoma. While CFRE may not require intervention, the extent of vascular and ureteral variation observed in this case has direct implications for surgical planning, including nephrectomy, transplantation, and urologic procedures. Such variation may complicate intraoperative management if unrecognized. This case highlights a unique presentation of CFRE Type E with significant anatomical complexity. Recognition of such variants may improve diagnostic accuracy and surgical preparedness, and their inclusion in anatomical education may enhance clinical awareness among trainees.

## Introduction

An ectopic kidney is a congenital renal anomaly where one or both kidneys assume an abnormal location. This often results from abnormal development or migration of the kidney(s). In normal fetal development, the kidneys will form in the pelvic region and ascend into the lumbar retroperitoneal space between approximately weeks 6-9 of gestation [1]. During this phase, the kidneys receive blood supply from the iliac vessels and, ultimately, from the abdominal aorta as it ascends. The transient embryologic vessels arising from the iliac arteries and veins typically regress once the kidneys reach their final position in the posterior abdominal wall at the upper lumbar level (roughly T12-L3 in the adult) [2].

Crossed fusion renal ectopia (CFRE) is a congenital anomaly in which one kidney crosses the midline during its ascent from the pelvis and fuses with the contralateral kidney. It is theorized that CFRE begins when the ureteric bud deviates toward the opposite metanephric blastema during the fourth to fifth week, initiating the midline crossing. In Type E variants, the kidneys likely fuse within the confined pelvic space, where mechanical pressure causes the crossed kidney to rotate into a transverse orientation before cephalad migration. As this fused unit ascends, it often fails to undergo standard vascular remodeling, retaining transient embryologic vessels from the iliac arteries and lower aorta that would typically regress. This lack of regression can result in complex morphology and persistent accessory vasculature [1,2].

The incidence of CFRE has been estimated at approximately one in 7,500 based on autopsy studies, and between one in 2,000 and one in 3,000 in live births. The condition shows a male predominance, with a reported male-to-female ratio of approximately 2:1-3:1 across studies [3].

CFRE is often asymptomatic and is typically discovered incidentally during imaging, surgical procedures, or autopsies. In rare cases when symptoms arise, the most common complaints are flank or abdominal pain, hematuria, or urinary tract infection; these may result from obstruction, urolithiasis, vesicoureteral reflux, or infection related to abnormal anatomy. It is speculated that flank or abdominal pain may be due to strain on the renal vessels from the weight of the kidney [4].

Crossed fusion renal ectopia is classified into six distinct subtypes, traditionally listed in decreasing order of clinical frequency: Type A (inferior crossed fusion), Type B (sigmoid kidney), Type C (lump kidney), Type D (disc kidney), Type E (L-shaped kidney), and Type F (superiorly crossed fused). This classification system is widely used in the literature and is publicly available for academic use [5].

This report documents a Type E (L-shaped) subtype. The L-shaped subtype has been reported in approximately 15-20% of CFRE cases, although reported frequencies vary considerably depending on the classification criteria used [6]. This subtype occurs when the crossed kidney rotates to a transverse position and fuses with the inferior pole of the contralateral kidney during its ascension [1]. Ths report highlights its morphology, medical relevance, and potential use in anatomical education.

## Case presentation

Dissection and kidney morphology

The variant kidney was discovered during a routine dissection of the retroperitoneal area on a formalin-fixed, whole-body anatomical donor, following Grant’s Dissector, 17th edition [7]. This donor’s cause of death was acute hypoxic respiratory failure at the age of 76 years. Comorbidities listed were hypertension, atrial flutter, hypothyroidism, aortic insufficiency, and high levels of panel reactive antibodies. The donor had a self-reported medical history of bilateral pleural effusions, bilateral bacterial pneumonia, and malignant renal cell carcinoma (RCC).

**Figure 1:**
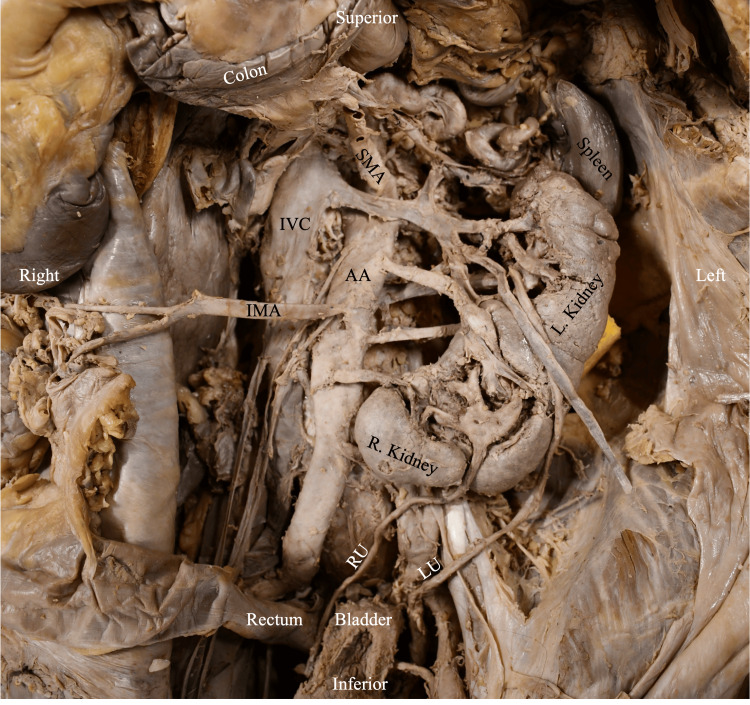
CFRE Type E kidney and surrounding structures in situ RU: right ureter; LU: left ureter; AA: abdominal aorta; SMA: superior mesenteric artery; IMA: inferior mesenteric artery; ICV: inferior vena cava; CFRE: crossed fused renal ectopia Renal classification and nomenclature adapted from: [4,5]

During the dissection of the retroperitoneal area, the inferior margin of the fused kidney was initially discovered where it crossed over the abdominal aorta, slightly superior to the common iliac bifurcation. After removal of the perinephric fat, examination revealed that the fused kidney was predominantly on the left posterior abdominal wall, with the right kidney considerably smaller than the left. The right kidney lay in a transverse orientation and was fused with the inferior-medial aspect of the left kidney. The right retroperitoneal area only contained retroperitoneal fat. This can be seen in Figure 1.

**Figure 2:**
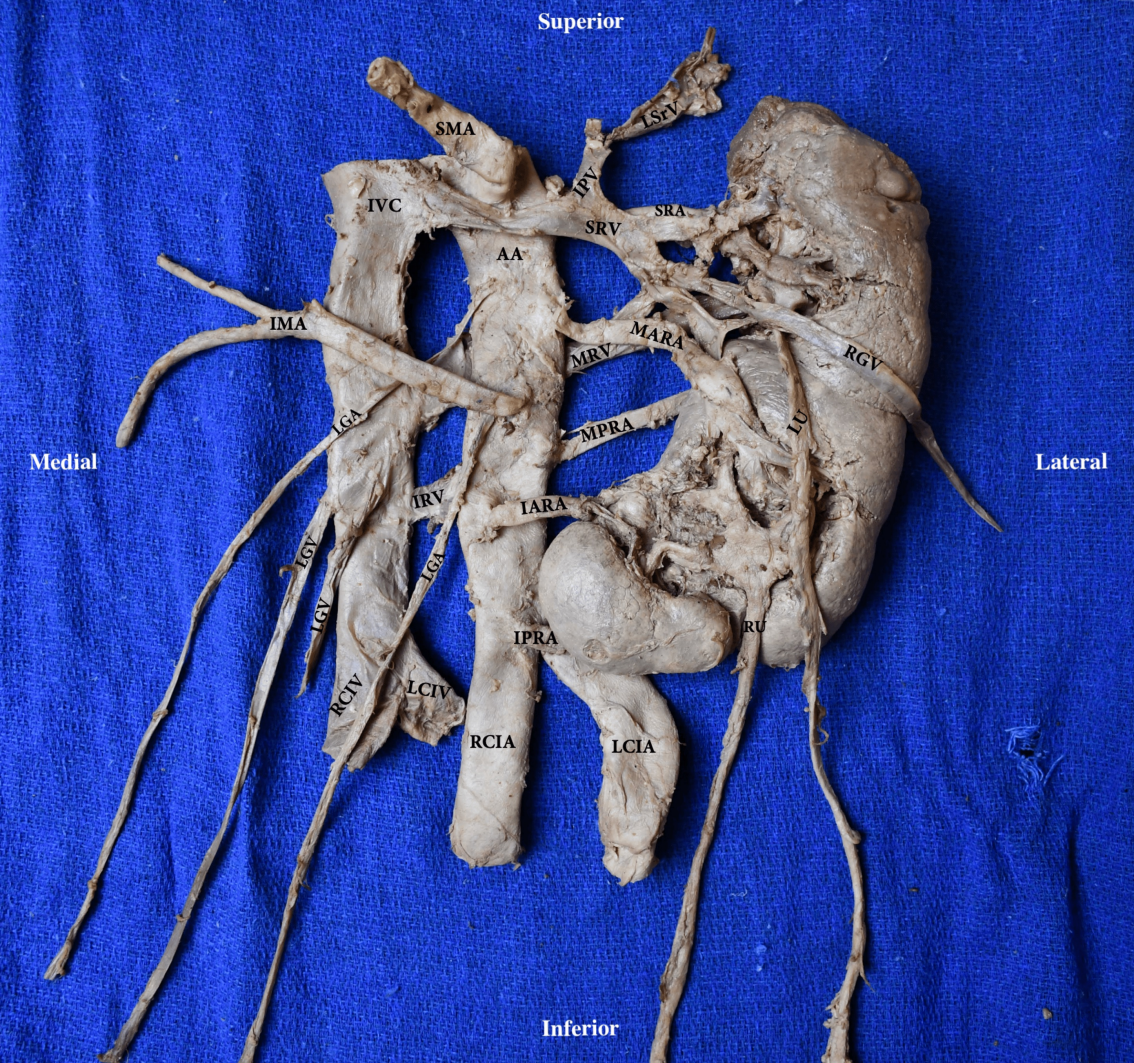
Anterior view of the CFRE Type E kidney, ureters, and vasculature ex situ RU: right ureter; LU: left ureter; AA: abdominal aorta; SMA: superior mesenteric artery; LGA: left gonadal artery; SR: superior renal artery; MARA: middle anterior renal artery; MPRA: middle posterior renal artery; IARA: inferior anterior renal artery; IPRA: inferior posterior renal artery; RCIA: right common iliac artery; LCIA: left common iliac artery; ICV: inferior vena cava; LGV: left gonadal vein; RGV: right gonadal vein; IPV: inferior phrenic vein; LSrV: left suprarenal vein; SRV: superior renal vein; MRV: middle renal vein; IRV: inferior renal vein; RCIV: right common iliac vein; LCIV: left common iliac vein; CFRE: crossed fused renal ectopia Renal classification and nomenclature adapted from: [4,5]

**Figure 3:**
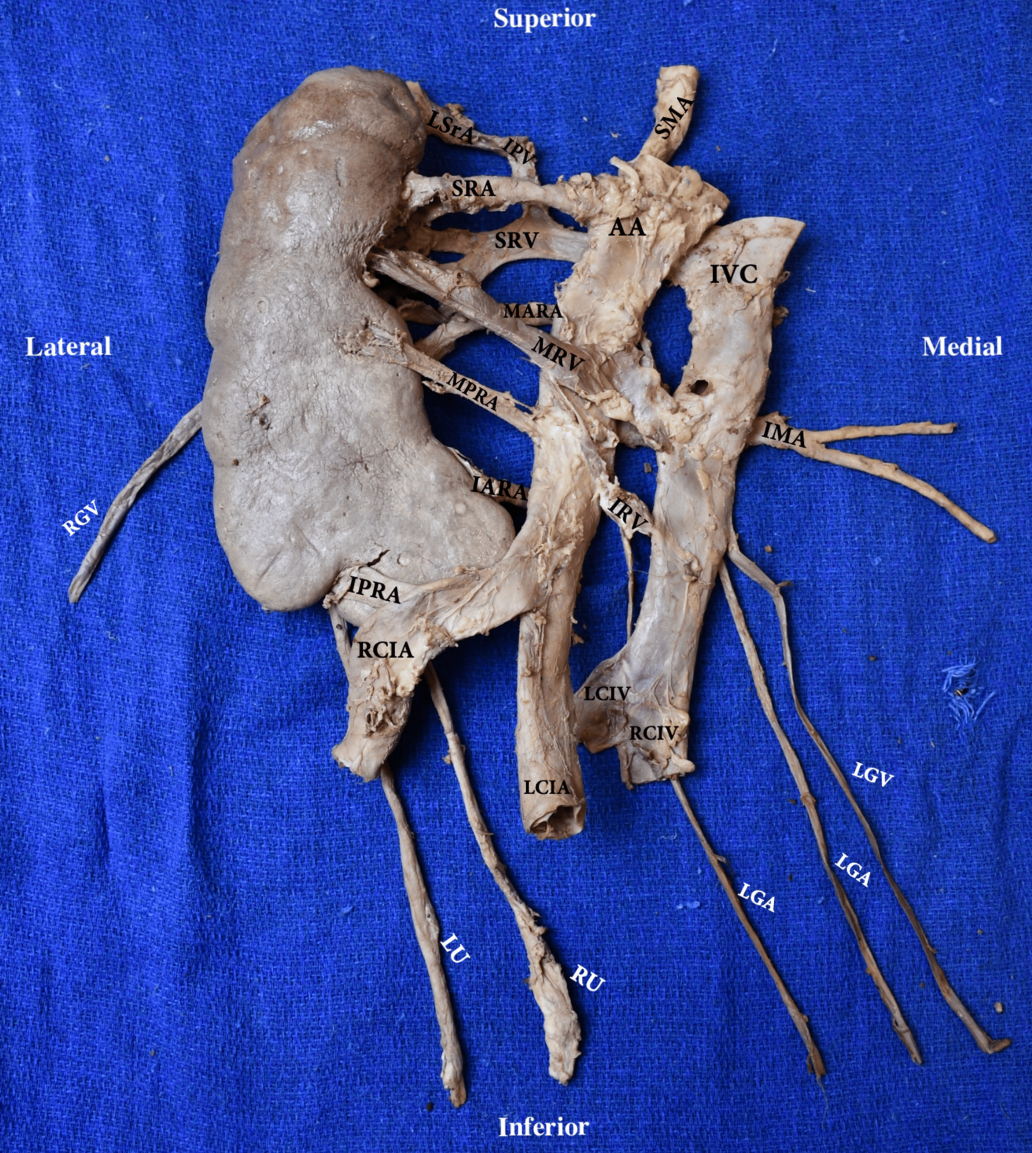
Posterior view of the CFRE Type E kidney, ureters, and vasculature ex situ RU: right ureter; LU: left ureter; AA: abdominal aorta; SMA: superior mesenteric artery; LGA: left gonadal artery; SR: superior renal artery; MARA: middle anterior renal artery; MPRA: middle posterior renal artery; IARA: inferior anterior renal artery; IPRA: inferior posterior renal artery; RCIA: right common iliac artery; LCIA: left common iliac artery; ICV: inferior vena cava; LGV: left gonadal vein; RGV: right gonadal vein; IPV: inferior phrenic vein; LSrV: left suprarenal vein; SRV: superior renal vein; MRV: middle renal vein; IRV: inferior renal vein; RCIV: right common iliac vein; LCIV: left common iliac vein; CFRE: crossed fused renal ectopia Renal classification and nomenclature adapted from: [4,5]

A raised outline of the right kidney was seen on the anterior surface, where it had fused with the medial aspect of the inferior pole of the left kidney. On the posterior side, there was no visible discrimination of where the right and left kidneys would have fused. This can be seen in Figures 2, 3.

**Figure 4:**
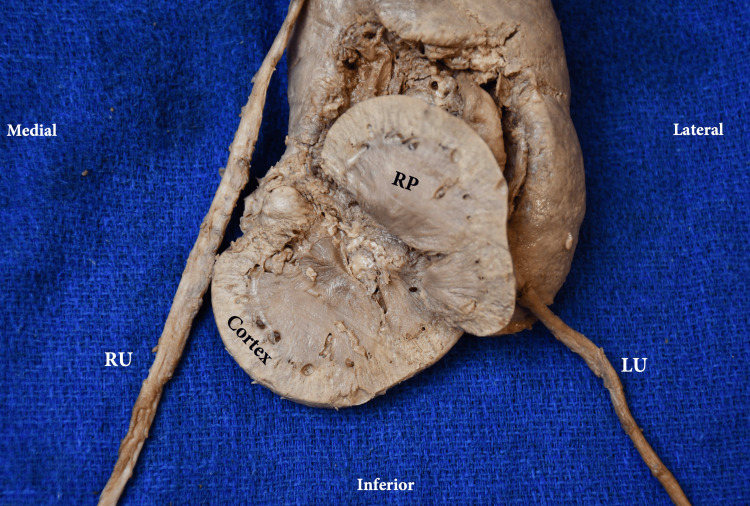
Right CFRE Type E kidney parenchyma (coronal section) RP: renal pyramid; RU: right ureter; LU: left ureter; CFRE: crossed fused renal ectopia Renal classification from: [5]

**Figure 5:**
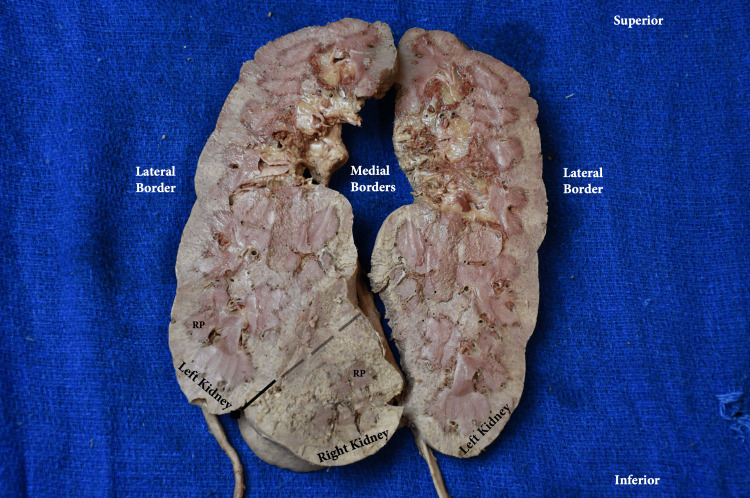
Right and left CFRE Type E kidney parenchyma (coronal section) RP: renal pyramid; CFRE: crossed fused renal ectopia Dashed line demarcates the left and right kidneys Renal classification from: [5]

Further dissection was done to expose the parenchyma of the right and left parts of the fused kidney. The smaller right kidney showed normal morphology, with the renal pyramids oriented toward the calyces (Figure 4). The larger, left-sided kidney exhibited a greater number of renal pyramids within the renal medulla. Many of these renal pyramids were irregular in shape and orientation (Figure 5). Dimensions, mass, and all other measurements of the kidney and vasculature are listed in Table 1.

**Table 1:** Measurements of CFRE Type E kidney and associated structures N/A: not applicable or not measured; CFRE: crossed fused renal ectopia Renal classification and nomenclature adapted from: [4,5]. Normal average values from: [8]

Structure	Ex situ measurements	Right	Left	Normal average values
Kidney	Length (superior to inferior pole, mm)	37.2	153.7	110-120
	Width (level of hilum, mm)	61.5	80.7	40
	Thickness (antero-posterior, mm)	34.6	25.6	20
	Mass (total, g)	N/A	223.7	150–200
Arteries (length, mm)	Superior renal a.	N/A	61.5	N/A
	Middle anterior renal a.	N/A	58.3	50
	Middle posterior renal a.	N/A	56.8	N/A
	Inferior anterior renal a.	N/A	48.7	N/A
	Inferior posterior renal a.	N/A	22.6	N/A
Veins (length, mm)	Superior renal v.	N/A	89.7	N/A
	Middle renal v.	N/A	101.7	70
	Inferior renal v.	N/A	58.5	N/A
Urinary Tract (width, mm)	Minor calyx (average)	7.2	9	N/A
	Major calyx (average)	10.8	13	N/A
	Renal pelvis	18.1	19.9	N/A
	Ureter	5.4	4.5	N/A

Arterial supply

**Figure 6:**
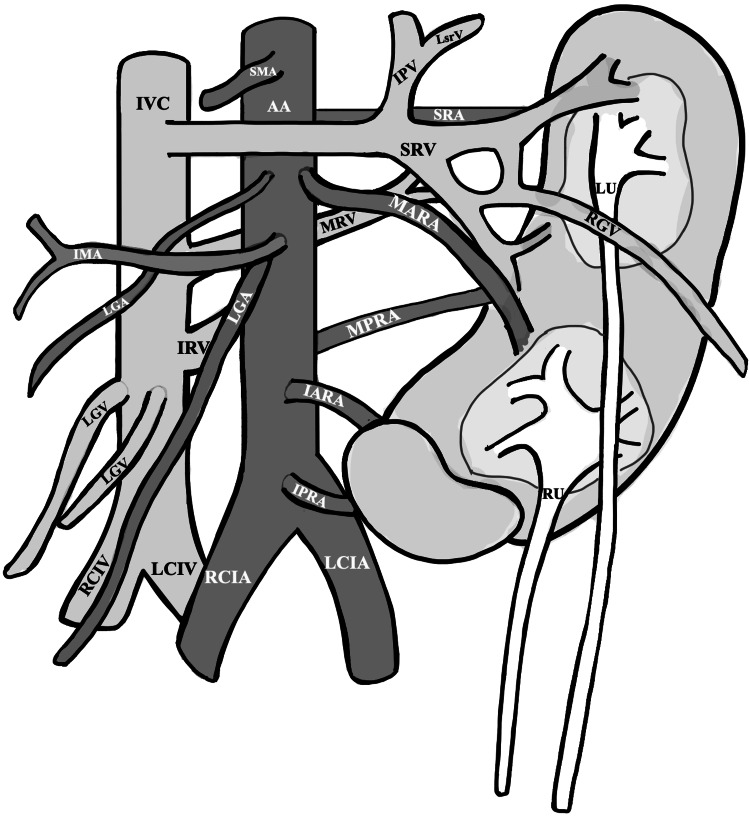
Line drawing of CFRE Type E kidney, ureters, and vasculature RU: right ureter; LU: left ureter; AA: abdominal aorta; SMA: superior mesenteric artery; LGA: left gonadal artery; SR: superior renal artery; MARA: middle anterior renal artery; MPRA: middle posterior renal artery; IARA: inferior anterior renal artery; IPRA: inferior posterior renal artery; RCIA: right common iliac artery; LCIA: left common iliac artery; ICV: inferior vena cava; LGV: left gonadal vein; RGV: right gonadal vein; IPV: inferior phrenic vein; LSrV: left suprarenal vein; SRV: superior renal vein; MRV: middle renal vein; IRV: inferior renal vein; RCIV: right common iliac vein; LCIV: left common iliac vein; CFRE: crossed fused renal ectopia Renal classification and nomenclature adapted from: [4,5]. Image Credit: Authors; created using Microsoft OneNote (Microsoft Corporation, Redmond, Washington, United States) [9]. No AI-assisted generation was used.

The regional vascular anatomy of the celiac trunk, superior mesenteric artery, inferior mesenteric artery, and iliac arteries was typical in structure. The arterial branches were descriptively named based on relative position and course, as no standardized nomenclature exists for such variants. There were five distinct arteries, from superior to inferior, that branched from the left side of the abdominal aorta and inserted into the parenchyma of the kidney. The superior renal artery (SRA) was the first branch, and it supplied the posterior aspect of the superior pole. The middle anterior renal artery (MARA) was the second branch, and it supplied the anterior aspect of the medial curvature, where the hilum would be in a non-variant kidney. The middle posterior renal artery (MPRA) was the third branch, and it supplied the posterior aspect of the medial curvature. The anterior inferior renal artery (IARA) was the fourth branch and supplied the anterior aspect of the inferior pole. The posterior inferior renal artery (PIRA) was the fifth branch, and it supplied the posterior aspect of the inferior pole. The right kidney received its blood supply from the IARA and PIRA; all of the other arteries supplied the larger left kidney. A simplified line drawing depicting the renal vasculature is shown in Figure 6.

Venous drainage

Three distinct renal veins were branching off the left side of the inferior vena cava: the superior renal vein (SRV), the middle renal vein (MRV), and the inferior renal vein (IRV). The SRV crossed the abdominal aorta anteriorly and displayed three merging tributaries, where two veins drained blood above the superior renal pelvis, and one vein drained below the same structure. The MRV coursed posterior to the abdominal aorta and merged with the inferior-most tributary of the SRV. The IRV crosses the posterior aspect of the abdominal aorta and attaches to the posterior aspect of the kidney, slightly inferior to the MPRA.

Urinary tract

There were two apparent renal pelvises, each forming a ureter that inserted into the bladder on either side. Both renal pelvises were on the anterior aspect and formed by the merging of three major calyces. The left renal pelvis was positioned near the superior pole of the left kidney. The ureter from this renal pelvis inserts on the left lateral side of the bladder. The right renal pelvis also emerges from the left kidney, but lateral to the inferior pole. A single major calyx can be seen exiting the right kidney and draining into the left renal pelvis, while the other two major calyces are coming from the left kidney. This ureter inserts into the right lateral side of the bladder. These are shown in Figure 6. The bladder showed no gross anatomical variation or pathology.

**Figure 7:**
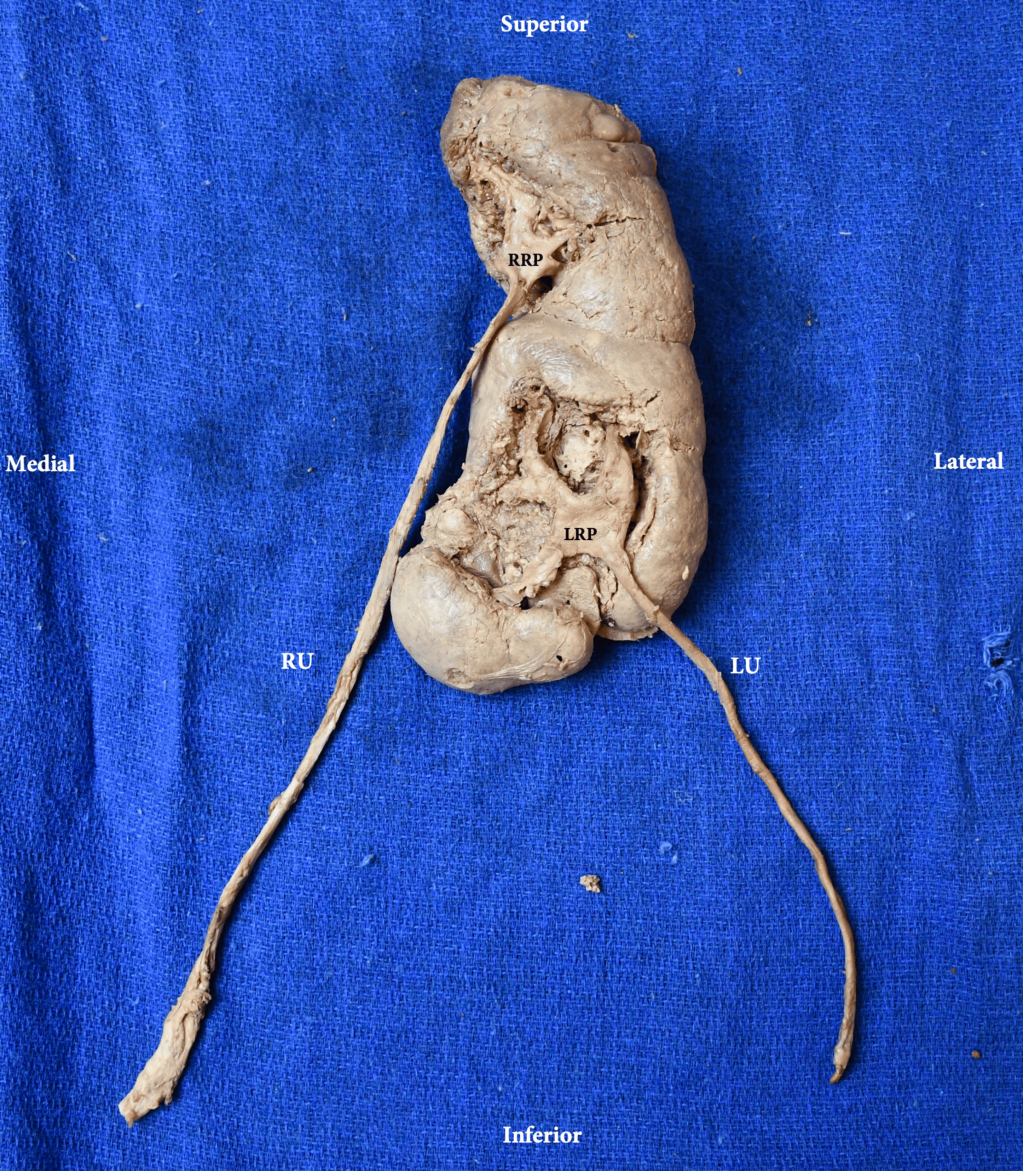
Anterior view of the CFRE Type E kidney and ureters ex situ RRP: right renal pelvis; LRP: left renal pelvis; RU: right ureter; LU: left ureter; CFRE: crossed fused renal ectopia Renal classification and nomenclature adapted from: [4,5]

## Discussion

Clinical and surgical relevance

Typically, CFRE remains undiagnosed unless it becomes symptomatic. The most frequently reported symptoms include flank or abdominal pain, dysuria, and hematuria [3]. When patients with CFRE become symptomatic, diagnostic tests typically include ultrasonography for quick visualization. This is often followed by CT angiography or MR angiography as a confirmatory test and for preoperative vascular mapping when surgery is planned [3,10].

The significant vascular variation observed in this case of CFRE could have direct implications for nephrectomy, renal transplantation, and urological procedures [11,12]. Our donor presented with five renal arteries and three renal veins, unlike other cases reporting fewer vessels, highlighting the need for detailed preoperative imaging, as vascular origins can range from the upper abdominal aorta to the iliac arteries [13]. Ureteral anomalies are also common in CFRE, with duplications, fused ureters, or a single draining ureter reported in the literature [10,12].

The donor’s history of RCC adds to the rarity of CFRE-associated malignancies; approximately 30 cases have been reported, with clear cell RCC being the most common histologic type [11,13]. Transperitoneal partial nephrectomy remains the favored surgical approach, but the complexity of CFRE anatomy makes each case uniquely challenging [13]. Because CFRE may coexist with other congenital anomalies, such as VACTERL (vertebral defects, anal atresia, cardiac defects, tracheo-esophageal fistula, renal anomalies, and limb abnormalities) and MURCS (müllerian duct aplasia, renal aplasia, and cervical/somite dysplasia) clinicians may consider targeted evaluation for associated anomalies guided by clinical history and exam, especially in children or when other signs suggest syndromic associations [10]. This case contributes valuable insight to the limited CFRE literature, especially in oncologic contexts.

Use in anatomical education

The use of donor-based anatomy is debated amongst medical schools and, to a lesser extent, undergraduate schools [14]. Additionally, anatomical variation is not always emphasized heavily in both programs, likely due to the volume and fast-paced nature of their respective curricula [15]. It has also been shown that anatomical variation is not often assessed in medical school, but it is widely assessed in postgraduate training and residency programs [16]. Because of this, it may be important to further incorporate anatomic variation into medical education, as it could benefit medical training beyond medical school.

There is variation in every anatomic structure, so it is not reasonable or feasible to teach every variant that may be encountered clinically or surgically. However, certain variants are common enough or sufficiently medically relevant to be included in routine education.

A study of 55 donors showed that 89% had two to five extra-laryngeal branches on the right and 75% had two to three on the left. This is clinically significant, as procedures such as thyroidectomy and anterior cervical discectomy and fusion (ACDF) can damage these branches, resulting in phonation deficits [17].

Another pertinent study shows that a lack of knowledge of femoral vein variation has led to increased iatrogenic vascular injuries during varicose vein surgery. With an injury incidence rate of 0.002-0.3% during varicose vein surgery, up to 11% of injuries are caused by the health care provider [18].

Although gross anatomy and embryology are explicit components of the United States Medical Licensing Examination (USMLE), the Comprehensive Osteopathic Medical Licensing Examination (COMLEX), and the Anatomical Society's Core Syllabus, variant anatomy is generally not listed as a separate, emphasized topic in those outlines; therefore, curricular emphasis on clinically relevant anatomic variation varies between schools [19-21]. Most medical schools teach basic and clinical science courses in accordance with these documents to best prepare their students for board examinations [15].

The ideal time to introduce and revisit medically significant variant anatomy has not yet been determined and remains under consideration [20]. Since anatomy is not traditionally taught in the clinical years of medical school (years 3-4), it may be best to introduce variant anatomy in the pre-clinical years (years 1-2) during the respective anatomy course. The selected anatomic variants can then be revisited, with potential clinical significance and/or surgical complications as they relate to future courses included. For example, our case of CFRE Type E could be showcased during the retroperitoneal or kidney lectures during an anatomy course as a possible anatomical variant, as it is something that could be seen on physical examinations or medical imaging. In future courses that discuss renal surgery or pathologies, this variant can again be shown, and a discussion could be held about the increased incidence of malignant renal cell carcinoma, potential surgical complications, or how to diagnose it using radiographic imaging.

Engaging with rare anatomical presentations, such as CFRE, during training reinforces the importance of patient-specific evaluation. Early exposure prepares future healthcare providers to navigate anatomical uncertainty with greater confidence and precision, bridging the gap between textbook anatomy and real-world clinical complexity [20].

## Conclusions

This congenitally abnormal CFRE kidney exhibits a unique morphology and is associated with a rare patient’s medical history. CFRE Type E is generally a benign condition that is often found incidentally, but it can pose challenges during clinical and surgical procedures due to its anatomical characteristics. This case is an example of anatomical variation that could be introduced in preclinical medical education. Knowledge and an understanding of this variant will aid medical professionals in their diagnostic and surgical awareness.
